# The Potential for Combined Treponemal/Nontreponemal Rapid Point-of-Care Test and *Treponema pallidum* Polymerase Chain Reaction in the Diagnosis of Gestational and Congenital Syphilis in a Low-Resource, High-Prevalence Setting: Pilot Data From Malawi

**DOI:** 10.1097/OLQ.0000000000002356

**Published:** 2026-05-15

**Authors:** Deirdre J Foley, Vita Nyasulu, Chifundo Kondoni, Annie Kuyere, Fatima Mtonga, George Shaba, James Jafali, Chelsea Morroni, Michael Marks, Patrick Mallon, David Lissauer, Gladys Gadama, Luis Gadama, Kondwani Kawaza, Charlotte van der Veer, Bridget Freyne

**Affiliations:** From the * School of Medicine, University College Dublin; † Departments of Infectious Diseases and Global Health, Children’s Health Ireland, Dublin, Ireland; ‡ Malawi Liverpool Wellcome Research Program, Blantyre, Malawi; § Centre for Reproductive Health, University of Edinburgh, Edinburgh, Scotland, United Kingdom; ¶ Botswana Harvard Health Partnership, Gaborone, Botswana; ∥ Clinical Research Department, Faculty of Infectious and Tropical Diseases, London School of Hygiene and Tropical Medicine; ** Hospital for Tropical Diseases, University College London Hospital, London, United Kingdom; †† Centre for Experimental Host Pathogen Research and Faculty of Paediatrics, University College Dublin, Dublin, Ireland; ‡‡ Institute of Life Course and Medical Sciences, University of Liverpool, Liverpool Women’s Hospital, Liverpool, United Kingdom; §§ Department of Obstetrics; ¶¶ John Hopkins Research Project; ∥∥ Department of Paediatrics, Queen Elizabeth Central Hospitaland; *** Department of Paediatrics, Kamuzu University of Health Sciences, Blantyre, Malawi

## Abstract

**Background:**

Congenital syphilis (CS) remains a major cause of stillbirth and neonatal morbidity, with an estimated 700,000 cases and 390,000 adverse birth outcomes annually. We evaluated the diagnostic utility of combined treponemal (TT) and nontreponemal (NTT) rapid point-of-care test and *Treponema pallidum* polymerase chain reaction (PCR) for detecting active maternal syphilis, CS, and treatment response in a high-prevalence, low-resource setting.

**Methods:**

Secondary analyses were conducted from a prospective case–control study at Queen Elizabeth Central Hospital, Malawi. Women were recruited ≤48 h postpartum. Maternal and infant sera underwent testing with the DPP® Syphilis Screen and Confirm (Dual rapid diagnostic test [RDT]) and *T. pallidum* PCR (maternal vaginal swabs and infant nasopharyngeal swabs), with predefined clinical-serological reference standards based on qualitative rapid plasma reagin (RPR).

**Results:**

Among 504 of 510 women with complete data, Dual RDT identified 110 seropositive cases at delivery, including 86 new maternal syphilis diagnoses. The NTT band showed good performance in mothers versus RPR (sensitivity: 84.8% [95% confidence interval: 77.4%–92.1%]; specificity 92.7% [95% confidence interval: 90.3%–95.1%]) but reduced sensitivity in infants, increasing from 51.9% to 80.0% with RPR titer. Laboratory visual and microreader interpretation showed high concordance (99.5%), while bedside visual accuracy was lower (77.4%). Any Dual RDT positivity identified 19 high-risk infants, of whom 7 of 19 (36.8%) were NTT positive. PCR detected maternal infection in 11 of 504 (2.2%), including 3 serology-negative cases.

**Conclusions:**

Dual RDT improves detection over TT alone. The NTT band without quantitation is insufficient for treatment monitoring or infant diagnosis. Combining Dual RDT with PCR may enhance detection of active maternal infection and CS in high-burden settings.

Congenital syphilis (CS) remains a leading cause of stillbirth and severe neonatal morbidity, with untreated maternal infection resulting in adverse birth outcomes (ABOs) in 50% to 70% of cases.^1^ In 2022, the World Health Organization (WHO) estimated there were 700,000 cases of CS, including 390,000 ABOs, with incidence in the African region 3-fold higher than the global average.^2^ WHO elimination of mother-to-child transmission strategy targets ≥95% antenatal screening and treatment coverage and a CS incidence <50 per 100,000 live births by 2030.^3^

WHO endorses screening strategies that prioritize nontreponemal testing (NTT), such as rapid plasma reagin (RPR), as a quantitative marker of disease activity, while acknowledging that this may not always be feasible and that an alternative approach based on treponemal-only rapid diagnostic tests (T-RDT) may be required.^4^ RPR implementation is resource-intensive, operator-dependent, and prone to interlaboratory variability with multifold titer discrepancies, risking over- and undertreatment. Additional limitations include false negatives in early or latent maternal infection.^5^ A 4-fold infant-to-maternal RPR rise occurs in only 10% to 55% of confirmed CS cases, and infant follow-up to attain confirmatory serology is logistically difficult with high attrition.^6,7^ Consequently, economic evaluations increasingly favor hybrid algorithms balancing coverage, overtreatment, and accurate identification of active infection.^8^

The HIV–syphilis testing coverage gap, the difference between people who get tested for HIV and those who get tested for syphilis, has reduced over the last decade, driven by improved availability of T-RDT, HIV/treponemal rapid diagnostic test (RDT), and triple RDT (with Hepatitis B surface antigen).^9,10^ Dual treponemal/nontreponemal RDT (Dual RDT) is supported by the WHO to improve same-day treatment, minimize loss to follow-up, and lower costs.^11^ A meta-analysis reported high diagnostic accuracy with treponemal components (treponemal test [TT]), achieving 93% sensitivity and 98% specificity, and nontreponemal (NT) components, achieving 90% sensitivity and 97% specificity, compared with laboratory-based reference standards.^12^ The DPP Syphilis Screen and Confirm specifically has reported a similar performance for its TT component and a sensitivity of 85% to 98.7% for the NT component, rising with RPR titer.^13^ Reader-assisted platforms (e.g., DPP® Microreader) convert visual signals into quantitative outputs approximating NT titers, reducing interobserver variability and correlating strongly with RPR titers.^14^ Implementation studies suggest improved workflow, reduced misclassification, and higher acceptability, although utility remains contingent on cost, supply chain reliability, and treatment availability.^15^ This is the first study to report on the performance of a Dual RDT in the diagnosis of CS.

An alternative to indirect, serological diagnostic tests is direct detection methods. Of these, *T. pallidum* polymerase chain reaction (PCR) has demonstrated 100% specificity and is considered confirmatory of CS within high-income country settings.^16,17^ In primary adult infection, PCR may detect pathogen DNA preseroconversion, with up to 10% of PCR-positive primary lesions being seronegative.^18^ PCR on nonlesion, mucosal samples has been shown to identify asymptomatic adult infection, with potential utility in asymptomatic infants.^19^ In confirmed CS, placental PCR has been reported as positive in 88% of cases, including all stillbirths (5/5), and nasal swab and cerebrospinal fluid PCR-positivity occurred in 4 of 7 (57%) and 2 of 8 (25%) tested infants, respectively.^6^ The sensitivity of whole-blood PCR reaches up to 83% (95% confidence interval [CI], 55.0%–95.2%) in infants with CS, although this meta-analysis included cord blood samples.^20^ Molecular diagnostics could potentially improve identification of high-risk asymptomatic infants and confirmation of symptomatic cases and stillbirths, although its implementation in this context is likely to be complex due to requirements for laboratory infrastructure, trained personnel, and stringent contamination control.

In Malawi, where syphilis seroprevalence among pregnant women is approximately 2%, antenatal testing is done using on-site T-RDT with same-day treatment.^21^ Malawian T-RDT scale-up has increased coverage but limits assessment of disease stage, treatment response, and CS diagnosis.^22^ The addition of Dual RDT, with or without molecular diagnostics, offers an attractive strategy to enhance screening, detect maternal reinfection, and refine infant risk stratification in high-burden, low-resource settings. In this study, we used available perinatal samples to pilot Dual RDT and *T. pallidum* PCR in assessing (1) maternal response to treatment during pregnancy, (2) maternal syphilis screening at delivery, (3) diagnosis of CS at birth, and (4) infant response to treatment.

## METHODS AND MATERIALS

Data were collected as part of a prospective case–control study of sexually transmitted infection prevalence and associated ABOs at Queen Elizabeth Central Hospital (QECH) in Blantyre, Malawi.^22^ All women who delivered an infant with an estimated gestation of 24 wk or greater at QECH were eligible for enrollment within 48 h postpartum. Enrollment occurred between August 2021 and March 2022. Maternal seropositivity was defined as antenatal T-RDT positivity and/or reactive RPR and/or TT-band positive Dual RDT at delivery. Maternal syphilis treatment adequacy was defined as ≥3 doses of intramuscular benzathine penicillin G (BPG) ≥30 d before delivery in line with national guidance. Maternal infection stage was classified using clinico-serological data, and infants were classified using the Center for Disease Control CS definitions criteria as outlined in Supplemental Digital Content 1, https://links.lww.com/OLQ/B371.^23^

Maternal blood at delivery was tested with RPR (Arkray, Kyoto, Japan) and DPP® Syphilis Screen and Confirm Dual RDT (Chembio, Medford, NY). RPR qualitative and quantitative results were independently interpreted by 2 laboratory technicians. In cases of discrepancies, a third technician performed an independent review to reach a final determination. For maternal and infant Dual RDT, we assessed the diagnostic accuracy, including sensitivity, specificity, positive predictive value, and negative predictive value, comparing the NTT to the laboratory-based RPR and associated CIs. Agreement between bedside and laboratory RDT results was reported using Cohen kappa and percent concordance. In addition, the balanced accuracy of the dual RDT band was calculated comparing (1) the microreader and visual readings in the laboratory (n = 564) and (2) the clinical team at the bedside and those read in the lab visually (n = 270). Infant sera and nasopharyngeal swabs (NPS) were collected from syphilis-exposed and selected unexposed infants, with repeat sera at 6-wk postdelivery where available. Maternal high vaginal swabs (HVS) and infant NPS were analyzed using *T. pallidum* polA-targeted singleplex PCR, a target that has demonstrated high diagnostic accuracy (sensitivity: 95.8%, specificity: 95.7%), using established primers (Integrated DNA Technologies, Coralville, IA).^24^ Cycle threshold (CT) ≤40 defined positivity, with independent review of CT value and control curves. Data were analyzed in Stata V. 13.1 (StataCorp LP, College Station, TX).

## RESULTS

**Figure 1.**
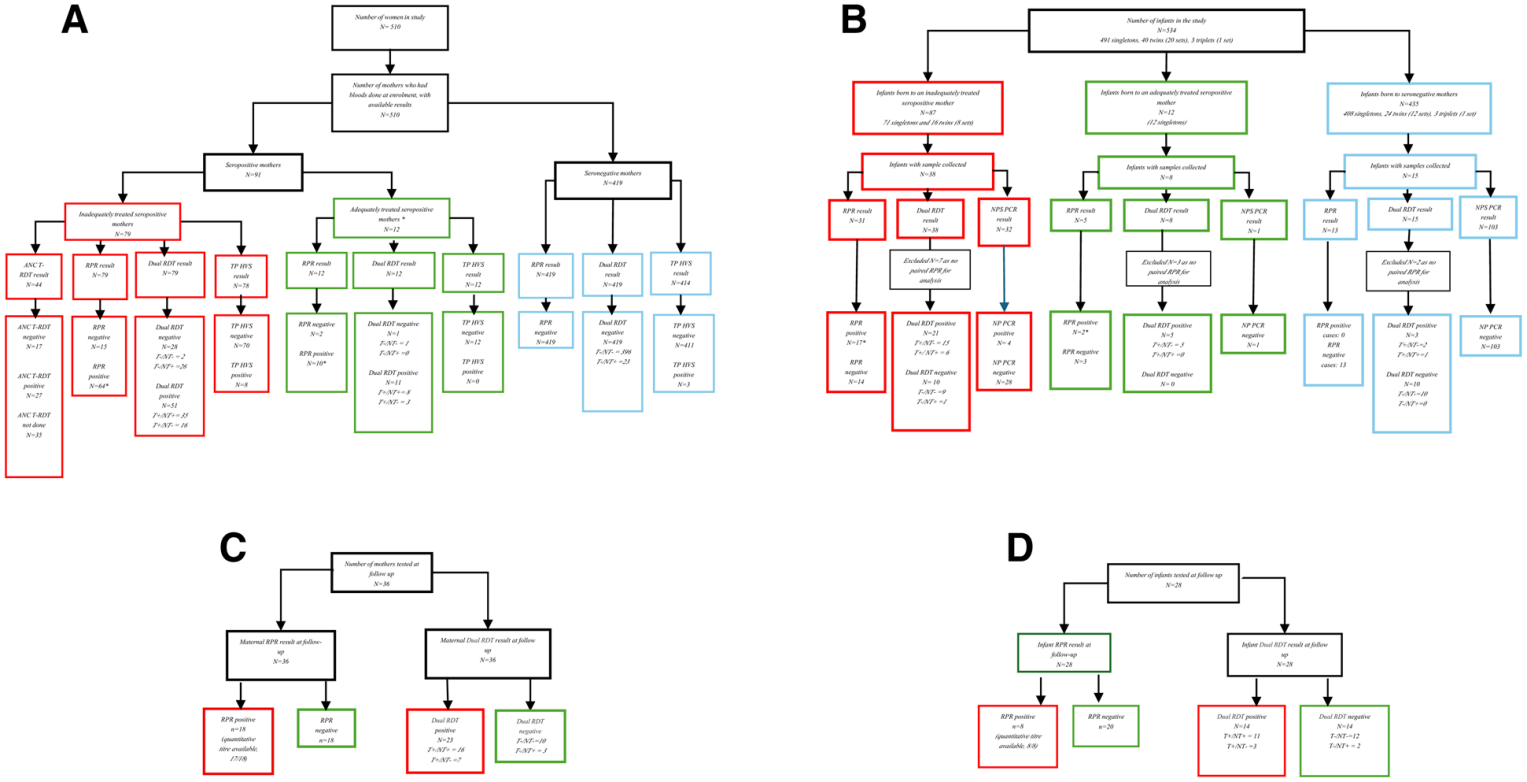
A, Maternal enrollment samples. B, Infant enrollment samples. C, Maternal follow-up samples. D, Infant follow-up samples.

Overall, 510 of 951 eligible women and their 534 infants were enrolled in the parent case–control study, of whom 91 women who delivered 99 infants were classified as seropositive for syphilis based on the definition outlined above (Fig. 1A–D). Of these, 27 had tested positive at antenatal care attendance (ANC), 29 had seroconverted between ANC and delivery, and 35 tested positive at delivery without a previously recorded syphilis test during ANC.

### Demographics

Among women who were seropositive for syphilis, the median age was 27 y, the prevalence of HIV was 28/91 (30.1%), and stillbirth occurred in 12/91 (13.2%). Among women who were seronegative for syphilis, the median age was 23 y, the prevalence of HIV was 58/419 (13.8%), and stillbirth occurred in 27/419 (6.4%). Among the 27 women diagnosed with syphilis during ANC screening, 23 received at least one dose of IM BPG. Of these, 7 received a single dose, 1 received 2 doses, and 15 completed 3 doses. Among women who received all 3 doses, 12 had their final dose administered at least 30 d before delivery (Table 1).

**TABLE 1.** Descriptive and Clinical Features of Mother–Infant Pairs Summarized by the Timing of Maternal Diagnosis

	T-RDT Positive at ANC (n = 27)	T-RDT negative at ANC/Seropositive at Delivery (n = 29)	No ANC Testing/Seropositive at Delivery (n = 35)
Maternal clinical characteristics			
Age, mean (SD), y	28.3 (8.1)	25.4 (7.2)	28.4 (6.4)
HIV positive	13 (48.2%)	2 (6.9%)	13 (37.1%)
HIV status unknown	1 (3.7%)	0 (0.0%)	2 (5.7%)
Adequately treated	12 (44.4%)	0	0
Maternal symptoms			
Painless genital ulcer	0/27 (0%)	2/29 (6.9%)	1/35 (2.9%)
Vaginal discharge	6/27 (22.2%)	2/29 (6.9%)	4/35 (11.4%)
Abdominal pain	3/27 (11.1%)	1/29 (3.5%)	1/35 (2.9%)
Dysuria	4/27 (14.8%)	1/29 (3.5%)	3/35 (8.6%)
None	14/27 (51.9%)	25/29 (86.2%)	29/35 (82.9%)
Infant clinical characteristics			
Total number of infants born	29	31	39
Stillborn infants	4/29 (13.8%)	3/31 (9.7%)	6/39 (15.4%)
Liveborn infants with ABO*	11/25 (44%) Premature and LBW, n = 4 LBW, n = 2 Premature, n = 1 HIE, n = 1 NICU admission, n = 3	15/28 (53.6%) Premature and LBW, n = 2 LBW, n = 5 Premature, n = 1 NICU admission, n = 7	20/33 (60.6%) Symptomatic CS, n = 1 Sepsis, n = 1 Premature and LBW, n = 2 LBW, n = 7 Premature, n = 4 NICU admission, n = 5
Liveborn asymptomatic infants	14/25 (56%)	13/28 (46.4%)	13/33 (39.4%)
WHO CS risk stratification of liveborn infants			
WHO CS, requiring treatment (liveborn infants)	15/25	28/28	33/33
CDC CS risk stratification of liveborn infants			
CDC confirmed or highly probable, n = 7	1	0	6
CDC possible, n = 25	7	11	7
CDC less likely, n = 11	11	0	0
CDC stage unknown, n = 43	6	17	20
IV benzylpenicillin indicated based on local guidelines	15/25	28/28	33/33

* ABO (symptomatic CS, premature [<37 wk], low birth weight [<2500 g], HIE, sepsis, admission to the NICU within 24 h), as classified by the case–control study.

ABO indicates adverse birth outcomes; CDC, Center for Disease Control; HIE, hypoxic–ischemic encephalopathy; NICU, neonatal intensive care unit; SD, standard deviation.

### Combined Dual RDT, RPR, and *T. pallidum* PCR in Identifying Active Maternal Syphilis at Delivery

About 504 of 510 women had a complete set of Dual RDT, HVS PCR, and RPR results available at delivery. Summary of maternal testing using the Dual RDT, RPR and PCR at point of delivery in women cohorted by T-RDT screening result (positive, negative or not done) are further outlined in Supplementary Digital Content 2.

#### RPR at Delivery

Overall, the maternal RPR was positive in 73 women at delivery. This included 21 women with a positive T-RDT during pregnancy and 52 new cases of maternal syphilis, 29 in those who were previously untested and 23 in those who tested negative at ANC (median titer 1:4, range 1:2 to 1:16).

#### Dual RDT at Delivery

One hundred and ten women were positive on one or more components of the Dual RDT at delivery. Of these, 24 had previously tested positive on a T-RDT during pregnancy; 21 were positive by RPR at delivery, of whom 16 were positive on both the NTT and TT components of the Dual RDT. The remaining 86 individuals were cases of syphilis newly identified through rescreening at delivery. This included 46 individuals who had seroconverted during pregnancy and 40 individuals who had not been tested during ANC care. Overall at delivery, 24/86 were TT+/NTT+, 14/86 were TT+/NTT−, and 48/86 were TT−/NTT+. Of 86 newly diagnosed individuals, 52 were RPR positive at delivery, of whom 47 individuals were also positive on the NTT component of the Dual RDT.

If any “NTT-band” positivity was designated a “true” positive, 91 women would be labeled with a diagnosis of syphilis, including 72/91 new cases (40/91 negative T-RDT, 32/91 unscreened at ANC). Sixty-three (63/91, 69.2%) of the NTT positive participants were also RPR positive. All RPR and PCR-positive women were identified when “any positive band” on the Dual RDT was used as a comparator.

#### *T. pallidum* PCR in Mothers and Infants at Delivery

There were 507 HVS collected at the point of delivery, and 504 available for *T. pallidum* PCR as part of this analysis, of which 11 (2.2%) were positive and 493 (97.8%) were negative. Of the 11/504 with active maternal syphilis confirmed by PCR, 7 were RPR positive at delivery, 6 were TT+/NTT+ and 1 TT−/NTT+. Four women had a positive PCR but negative RPR, 3 were negative on Dual RDT and 1 TT+/NTT+. Of those 11 women with positive HVS PCR, 2 were T-RDT positive but untreated at ANC screening, 3 were negative on T-RDT screening, and 6 were unscreened at ANC.

Among the 91 syphilis seropositive mothers, 12 were adequately treated in pregnancy, and all had a negative HVS PCR (Fig. 1A). Of 79 seropositive but inadequately treated mothers, 63 were RPR positive, of whom 7 (8.9%) had a positive HVS PCR. HVS PCR did not identify 56 women who were both RPR positive and were antenatally diagnosed but inadequately treated for syphilis in pregnancy or unscreened and subsequently positive at delivery.

Of 136 infant NPS samples, 4 (2.9%) infants were positive and 132 (97.1%) negative for *T. pallidum* PCR (Fig. 1B). There were 87 infants born to seropositive women who received inadequate treatment in pregnancy; 32/87 had an NPS for PCR, of which 4/32 (12.5%) were PCR positive (CT values range, 26.6–31.7). Clinical phenotypes included 1 case with classic symptoms of CS, 2 unwell infants (sepsis and hypoxic–ischemic encephalopathy), and 1 premature neonate (Supplemental Digital Content 5, https://links.lww.com/OLQ/B372). Three of the PCR-positive infants had paired maternal–infant RPR titers, none demonstrating a 4-fold discordance. All PCR-positive infants with Dual RDT data exhibited reactive TT bands, and 2 showed additional NTT-band reactivity. One infant born to an unscreened mother at ANC had no available serology and was classified as confirmed CS based on PCR detection alone. Of 132 infants with a negative *T. pallidum* PCR, 29 were syphilis-exposed and 103 were syphilis-unexposed (Fig. 1B). Among 16/29 syphilis-exposed, PCR-negative infants had an available RPR result; 8 were RPR negative and 8 had a positive RPR (range 1:4 to 1:16). Two infants with CS highly probable, who had a 4-fold rise in maternal–infant RPR, had a negative NPS PCR.

### Dual RDT NT Band Compared With Reference Standard (RPR) in Mothers and Infants, in the Laboratory and at the Bedside

Five hundred and forty six maternal Dual RDT tests were performed on 510 women in the study at baseline (n = 510) and at follow-up (n = 36). Seventy-seven infant Dual RDT tests were available for analysis on infants at baseline (n = 49) and at follow-up (n = 28). The overall sensitivity and specificity of the NTT band of the Dual RDT in mothers were 84.8% (95% CI, 77.4%–92.1%) and 92.7% (95% CI, 90.3%–95.1%), respectively. In infants, the sensitivity and specificity were 51.9% and 86%, respectively. Test performance varied according to the reference RPR titer, with sensitivity rising to 100% at RPR titer ≥1:16, and infant NT-band sensitivity rising similarly from 51.9% to 80% at RPR titer ≥1:16 (Table 2). Specificity decreased slightly, while positive predictive value declined markedly in both groups with increasing RPR titers. Laboratory visual T-RDT interpretation showed high concordance with microreader results (99.5% accuracy), versus lower bedside–laboratory agreement (77.4%) (Table 2).

**TABLE 2.** Specificity, Sensitivity, NPV, PPV, and Balanced Accuracy of the NT Band of the Dual RDT Compared With RPR

Performance of Whole-Blood NTT Band of Dual RDT in Mothers and Infants									
		+	−	Total	Sensitivity	Specificity	PPV	NPV	Accuracy
Whole-blood Dual RDT (NTT band) vs RPR in mothers									
Reference method	Test method: maternal whole-blood Dual RDT NTT band, laboratory (n = 546)								
Maternal RPR qualitative positive	+	78	33	111	84.8% (95% CI, 77.4%–92.1%)	92.7% (95% CI, 90.3%–95.1%)	70.3% (95% CI, 61.8%–78.8%)	96.8% (95% CI, 95.1%–98.4%)	88.8%
	−	14	421	435					
	Total	92	544	546					
Maternal RPR ≥ 1:8	+	32	79	111	86.5% (95% CI, 75.5%–97.5%)	84.5% (95% CI, 81.3%–87.6%)	28.8% (95% CI, 20.4%–37.3%)	98.9% (95% CI, 97.8%–99.9%)	85.5%
	−	5	430	435					
	Total	37	509	546					
Maternal RPR ≥ 1:16	+	11	100	111	100% (95% CI, 100%–100%)	81.3% (95% CI, 78.0%–84.6%)	9.9% (95% CI, 4.4%–15.5%)	100% (95% CI, 100%–100%)	90.7%
	−	0	435	435					
	Total	11	535	546					
Whole-blood Dual RDT (NT band) vs RPR in infants									
Reference method	Test method: infant whole-blood Dual RDT NTT band, laboratory (n = 77)								
Infant RPR qualitative positive	+	14	7	21	51.9% (95% CI, 33.0%–70.7%)	86% (95% CI, 76.4%–95.6%)	66.7% (95% CI, 46.5%–86.8%)	76.8% (95% CI, 65.7%–87.8%)	68.9%
	−	13	43	56					
	Total	27	50	77					
Infant RPR positive ≥ 1:8	+	7	14	21	77.8% (95% CI, 50.6%–100%)	79.4% (95% CI, 69.8%–89%)	33.3% (95% CI, 13.2%–53.5%)	96.4% (95% CI, 91.6%–100%)	78.6%
	−	2	54	56					
	Total	9	68	77					
Infant RPR positive ≥ 1:16	+	4	17	21	80% (95% CI, 44.9%–100%)	76.4% (95% CI, 66.6%–86.2%)	19% (95% CI, 2.3%–35.8%)	98.2% (95% CI, 94.7%–100%)	78.2%
	−	1	55	56					
	Total	5	72	77					
Whole-blood Dual RDT (NT band), laboratory visual vs microreader in mothers and infants									
Reference method	Test method: maternal whole-blood Dual RDT NTT band, laboratory visual (n = 564)								
Maternal whole-blood Dual RDT NTT band, laboratory microreader	+	100	1	101	99% (95% CI, 97.1%–100%)	100% (95% CI, 100%–100%)	100% (95% CI, 100%–100%)	99.8% (95% CI, 99.4%–100%)	99.5%
	−	0	463	463					
	Total	100	464	564					
Whole-blood Dual RDT (NT band) at beside vs laboratory in mothers and infants									
Reference method	Test method: combined maternal and infant whole-blood Dual RDT NTT band, bedside visual (n = 270)								
Maternal and infant whole-blood Dual RDT NTT band, laboratory visual	+	22	7	29	57.9% (95% CI, 42.2%–73.6%)	97.0% (95% CI, 94.8%–99.2%)	75.9% (95% CI, 60.3%–91.4%)	93.4% (95% CI, 90.2%–96.5%)	77.4%
	−	16	225	241					
	Total	38	232	270					

NPV indicates negative predictive value; PPV, positive predictive value.

#### Dual RDT to Monitor Maternal and Infant Response to Treatment

Twenty-eight women who tested positive for syphilis in pregnancy or delivery had paired Dual RDT and RPR results available at delivery and 6-wk follow-up (Fig. 1C). Notably, 18/28 (64.3%) received treatment with 1 dose of IM BPG (Supplemental Digital Content 3, https://links.lww.com/OLQ/B373), and the remainder had no documented treatment. Of 5 women who were RPR positive at delivery and RPR negative posttreatment, 2 seroreverted from NTT+ to NTT−, 2 remained NTT+, 1 remained NTT−, and 1 seroconverted from NTT− to NTT+.

Twenty-eight infants had follow-up bloods taken, 13 of whom had paired Dual RDT and RPR results at both delivery and 6-wk follow-up. Notably, 10/13 were born to mothers who had received inadequate antenatal treatment. Infant treatment was documented in only one of 13 (Supplemental Digital Content 4, https://links.lww.com/OLQ/B374). Five infants were RPR positive at delivery, of whom 2 became RPR negative at follow-up and 3 demonstrated a decline in RPR titer. Four infants with a positive RPR at birth were TT+/NTT−, of whom 3 became TT+/NTT+ at follow-up despite a reduction or seroreversion in RPR.

#### Dual RDT and PCR in Mothers of Stillborn Infants

**Figure 2.**
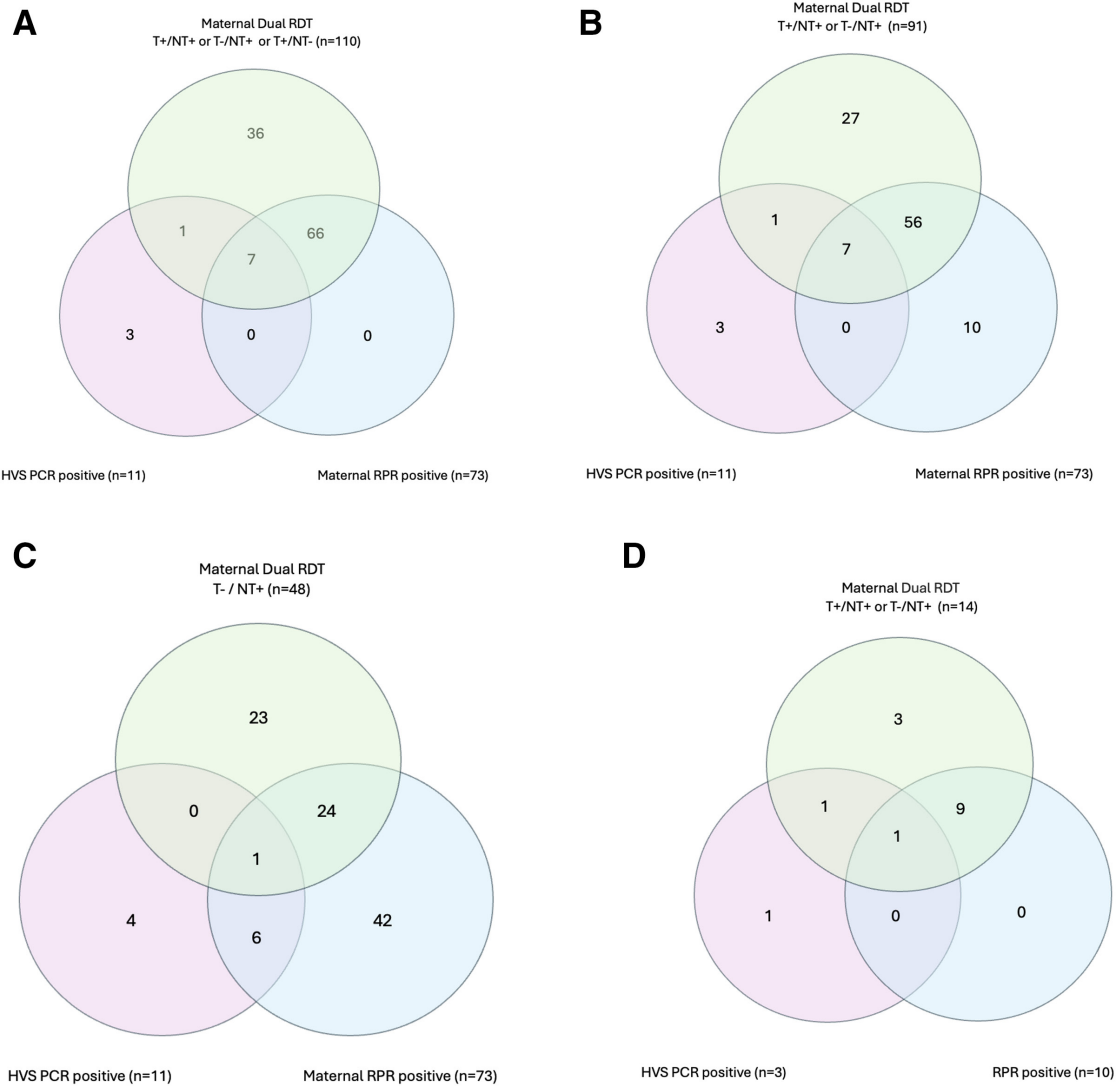
A–C, Venn diagrams representing the distribution and overlap of test positivity using the Dual RDT, HVS T. pallidum PCR, and serum RPR in women tested at the point of delivery using 3 different versions of Dual RDT positivity as a comparator: (A) any component positive; (B) any NTT-band positive; (C) NTT-band positive alone. D, Maternal Dual RDT, HVS T. pallidum PCR, and serum RPR results in mothers of stillborn infants.

There were 44/534 (8.2%) infants stillborn to 39 mothers, of whom 37/39 had combined Dual RDT, RPR, and PCR results available. Of these women, 14/37 had at any band positivity on Dual RDT (NTT+ only: n = 6, TT+/NTT+: n = 8). Ten were RPR positive. Three women were PCR positive, two of whom were RPR negative (TT+/NTT+: n = 1, TT−/NTT−: n = 1). Three infants were stillborn to women with negative RPR and PCR, but a positive NTT band. Maternal rescreening with Dual RDT at delivery identified 14 seropositive mothers of stillborn infants. The combination of Dual RDT and *T. pallidum* PCR identified all seropositive mothers of stillborn infants (Fig. 2).

### Dual RDT for the Identification of CS at Delivery Using 3 CS Reference Standards

Using infant clinical and diagnostic information as a comparator, 19 infants were classified as at-risk of CS based on naso-pharyngeal *T. pallidum* PCR-positivity, reactive RPR, and/or symptoms of CS. All had paired RPR and Dual RDT results available. The Dual RDT was positive on at least one band in all cases; 18/19 (94.7%) were TT-band positive and 7/19 (36.8%) had a positive NTT-band component. Among 17 infants classified as CS less likely (asymptomatic, RPR negative, and PCR-negative), none had a positive NTT band, while 8/17(47%) were TT-band positive (Supplemental Digital Content 6, https://links.lww.com/OLQ/B375).

Using paired maternal–infant RPR titer and T-RDT, 19 mother–infant pairs with inadequate maternal treatment and reactive RPR were classified as Center for Disease Control CS confirmed/probable (n = 6), CS possible (n = 11), and CS less likely (n = 2). All confirmed/probable cases were associated with maternal NTT positivity. One infant with a 4-fold rise in RPR was TT−/NTT+ in both mother and infant, indicating that reliance on treponemal testing alone would have missed this case. All infants classified as possible CS in this analysis were TT positive, with 3/11 also NTT positive. None of the CS less likely infants had a positive NTT component (Supplemental Digital Content 7, https://links.lww.com/OLQ/B376).

Using combined maternal history and infant diagnostics, 19 mother–infant pairs had complete data. Dual RDT was positive in all infants with reactive RPR and/or PCR-positivity. However, the NTT component failed to detect 4/19 (21%) higher-risk infants, including one PCR-positive case. Stratified analysis showed “any” Dual RDT positivity identified all higher-risk (10/11 via TT-band alone), while most lower-risk infants (7/8) were dual RDT negative (Supplemental Digital Content 8, https://links.lww.com/OLQ/B377).

## DISCUSSION

In this evaluation of a Dual RDT at the time of delivery, we found the test demonstrated robust sensitivity and specificity in mothers, consistent with prior reports.^13,25^ In infants, the NTT band demonstrated poor sensitivity compared with RPR, which improved with increasing titers. The NTT band alone did not reliably identify infants with CS. When “any component positive” was used, the Dual RDT identified all infants classified as high risk, regardless of the diagnostic framework applied, while all low-risk infants were NTT-band negative. In principle, the use of dual RDT with the potential for quantitative assessment of NT titer dynamics may improve diagnostic accuracy and utility in both response to treatment and infant risk assessment and warrant further evaluation. While evaluation of treatment response was limited to a small population, discordance between RPR seroreversion and persistent NTT-band positivity in both mothers and infants suggests the NTT band cannot reliably indicate posttreatment serological response.

Although third-trimester screening is recommended in high-burden settings, implementation remains inconsistent and guidance on optimal diagnostic strategies is limited.^26,27^ This study highlights important gaps in the interpretation of Dual RDT results, particularly isolated NTT-band positivity. One-third of RPR-positive women had isolated NTT-band positivity and could have been missed by T-RDT-only rescreening strategies.^25^ Data on Dual RDT from a controlled laboratory setting demonstrated that the Dual RDT NTT band identified 23/40 (57%) of RPR-positive serum samples on visual inspection of the test cartridge, increasing to 40/40(100%) on application of the DPP microreader, suggesting that NTT-band positivity may identify a subset of active infections but could lack specificity when used in isolation.^28^ Its clinical significance in routine care, therefore, remains uncertain and requires further evaluation incorporating quantitative measures, IgM-based assays, and clinical context.^29^

In addition to novel serological tests, we also assessed PCR as an adjunctive test for the detection of active syphilis in women. In this cohort, HVS PCR identified 3 maternal infections not evident on RPR and Dual RDT at delivery, supporting its potential role in detecting active infection before seroconversion. *T. pallidum* PCR at delivery also improved attribution of stillbirth to syphilis in this cohort, particularly when combined with Dual RDT. This has important implications for surveillance and evaluation of elimination of mother-to-child transmission progress, where accurate attribution of syphilis-related stillbirth remains a key gap.^30^ Our finding that *T. pallidum* PCR was more likely to be positive in infants with some symptoms and not necessarily in those with serologically confirmed disease is in keeping with the limited available data in this population.^7^ In neonates, *T. pallidum* PCR identified a premature infant with no clinical signs of infection and 2 unwell infants whose symptoms were not attributed to syphilis. These findings highlight the clinical heterogeneity of CS and suggest that PCR may be useful in selected phenotypes. The optimal sample type for neonatal PCR remains uncertain; adult data report improved yield with multisite sampling.^29^ In infants, reported sensitivities vary by specimen type, with estimates of 83% for blood and 62.2% for cerebrospinal fluid.^20^

To our knowledge, this is the first study to evaluate the combined use of Dual RDT and *T. pallidum* PCR in a perinatal cohort of mothers and infants. Limitations include the small number of infant samples, incomplete follow-up, and reliance on visual interpretation of Dual RDT results without microreader interpretation at the bedside. Additionally, the absence of RPR data from ANC may have resulted in misclassification of maternal–infant pairs. Our findings support the concept of integrated near-patient diagnostic strategies to improve identification of active infection in mothers, to refine infant risk stratification, and to reduce overtreatment associated with treponemal-only screening. The combination of Dual RDT with molecular diagnostics provides an attractive suite of tools for evaluation in this setting. Future studies should evaluate streamlined diagnostic algorithms incorporating minimum clinical data and targeting high-risk groups, including women at risk of reinfection and asymptomatic infants at risk of progression. Development of near-patient diagnostics must prioritize affordability and feasibility across healthcare settings and rigorous end-user and implementation evaluation before scale-up.

## Supplementary Material

## References

[1] Gomez GB Kamb ML Newman LM. Untreated maternal syphilis and adverse outcomes of pregnancy: A systematic review and meta-analysis. Bull World Health Organ 2013;91:217–226. 23476094 10.2471/BLT.12.107623 PMC3590617

[2] World Health Organization. Mother-to-child transmission of syphilis. Health topics - Global sexually transmitted infections programme. 2022. Available at: https://www.who.int/teams/global-hiv-hepatitis-and-stis-programmes/stis/prevention/mother-to-child-transmission-of-syphilis. Accessed June 6, 2026.

[3] World Health Organization. The global elimination of congenital syphilis: Rationale and strategy for action. 2007. Available at: https://www.who.int/publications/i/item/the-global-elimination-of-congenital-syphilis-rationale-and-strategy-for-action. Accessed February 3, 2026.

[4] World Health Organization. WHO guideline on syphilis screening and treatment for pregnant women. Geneva, Switzerland: World Health Organization; 2017. Available at: https://www.who.int/publications/i/item/9789241550093. Accessed September 9, 2025. 29757595

[5] Berkowitz K Baxi L Fox HE. False-negative syphilis screening: The prozone phenomenon, nonimmune hydrops, and diagnosis of syphilis during pregnancy. Am J Obstet Gynecol 1990;163:975–977. 2403176 10.1016/0002-9378(90)91107-n

[6] Lim SMJ Gooding H Walczak A. Improving the detection of congenital syphilis: Reviewing test utility and adherence to recommendations. Pathology (Phila) 2025;57:352–356. 10.1016/j.pathol.2024.09.010 39674694

[7] Garel B Grange P Benhaddou N. Congenital syphilis: A prospective study of 22 cases diagnosed by PCR. Ann Dermatol Venereol 2019;146:696–703. 31558291 10.1016/j.annder.2019.08.007

[8] Terris‐Prestholt F Vickerman P Torres‐Rueda S. The cost‐effectiveness of 10 antenatal syphilis screening and treatment approaches in Peru, Tanzania, and Zambia. Int J Gynecol Obstet 2015;130:73–80. 10.1016/j.ijgo.2015.04.007 PMC4510253 25963907

[9] Storey A Seghers F Pyne-Mercier L. Syphilis diagnosis and treatment during antenatal care: The potential catalytic impact of the dual HIV and syphilis rapid diagnostic test. Lancet Glob Health 2019;7:e1006–e1008. 31303285 10.1016/S2214-109X(19)30248-7 PMC6759458

[10] World Health Organization. WHO prequalifies the first triple diagnostic test for HIV, hepatitis B and syphilis, a milestone toward global disease elimination goals. Departmental Update. 2025.

[11] WHO. Updated recommendations for the treatment of *Neisseria gonorrhoeae*, *Chlamydia trachomatis* and *Treponema pallidum* (syphilis), and new recommendations on syphilis testing and partner services. Geneva, Switzerland: World Health Organization; 2024. 39137269

[12] Zhang Y Goh SM Mello MB. Improved rapid diagnostic tests to detect syphilis and yaws: A systematic review and meta-analysis. Sex Transm Infect 2022;98:608–616. 36180209 10.1136/sextrans-2022-055546 PMC9685714

[13] Marks M Yin YP Chen XS. Metaanalysis of the performance of a combined treponemal and nontreponemal rapid diagnostic test for syphilis and yaws. Clin Infect Dis 2016;63:627–633. 27217216 10.1093/cid/ciw348 PMC4981758

[14] Vargas SK Qquellon J Vasquez F. Laboratory evaluation of the DPP Syphilis Screen & Confirm assay. Microbiol Spectr 2022;10:e0264221. 35638776 10.1128/spectrum.02642-21 PMC9241612

[15] García PJ Cárcamo CP Chiappe M. Rapid syphilis tests as catalysts for health systems strengthening: A case study from Peru. PLoS One 2013;8:e66905. 23840552 10.1371/journal.pone.0066905 PMC3694115

[16] Grange PA Gressier L Dion PL. Evaluation of a PCR test for detection of *Treponema pallidum* in swabs and blood. J Clin Microbiol 2012;50:546–552. 22219306 10.1128/JCM.00702-11 PMC3295187

[17] Herremans T Kortbeek L Notermans DW. A review of diagnostic tests for congenital syphilis in newborns. Eur J Clin Microbiol Infect Dis 2010;29:495–501. 20336337 10.1007/s10096-010-0900-8

[18] Hughes Y Towns JM Ong JJ. The proportion of *Treponema pallidum* polymerase chain reaction–positive primary syphilis infections that are seronegative for syphilis: A systematic review and meta-analysis. Open Forum Infect Dis Oxford University Press 2025;12:ofaf471. 10.1093/ofid/ofaf471 PMC12461845 41018698

[19] Aung ET Fairley CK Williamson DA. *Treponema pallidum* detection at asymptomatic oral, anal, and vaginal sites in adults reporting sexual contact with persons with syphilis. Emerg Infect Dis 2023;29:2083–2092. 37703891 10.3201/eid2910.230660 PMC10521609

[20] Gayet-Ageron A Ninet B Toutous-Trellu L. Assessment of a real-time PCR test to diagnose syphilis from diverse biological samples. Sex Transm Infect 2009;85:264–269. 19155240 10.1136/sti.2008.034314

[21] Chirombo J Majamanda A Gunsaru V. The prevalence of gestational syphilis in Malawi between 2014 and 2022: Spatiotemporal modeling of population-level factors. Front Public Health 2024;11:1242870. 38292384 10.3389/fpubh.2023.1242870 PMC10825961

[22] van der Veer C Kondoni C Kuyere A. Prevalence of sexually transmitted infection in pregnancy and their association with adverse birth outcomes: A case–control study at Queen Elizabeth Central Hospital, Blantyre, Malawi. Sex Transm Infect 2024;100:517–523. 39043612 10.1136/sextrans-2024-056130 PMC11671869

[23] CDC. Congenital syphilis. Sexually transmitted infections treatment guidelines. 2021. Available at: https://www.cdc.gov/std/treatment-guidelines/congenital-syphilis.htm. Accessed November 11, 2025.

[24] Liu H Rodes B Chen CY. New tests for syphilis: Rational design of a PCR method for detection of *Treponema pallidum* in clinical specimens using unique regions of the DNA polymerase I gene. J Clin Microbiol 2001;39:1941–1946. 11326018 10.1128/JCM.39.5.1941-1946.2001 PMC88053

[25] Langendorf C Lastrucci C Sanou-Bicaba I. Dual Screen and Confirm Rapid test does not reduce overtreatment of syphilis in pregnant women living in a non-venereal treponematoses endemic region: A field evaluation among antenatal care attendees in Burkina Faso. Sex Transm Infect 2019;95:402–404. 30580325 10.1136/sextrans-2018-053722 PMC6824609

[26] Desjardins AA Amaral E Miranda J; FIGO Committee on Infections During Pregnancy and FIGO Committee on Health Systems Strengthening. Syphilis in pregnancy: A practical guide for prenatal care providers. Int J Gynaecol Obstet 2025;171:601–610. 40977496 10.1002/ijgo.70511 PMC12553112

[27] Papp JR Park IU Fakile Y. CDC laboratory recommendations for syphilis testing, United States, 2024. MMWR. Recomm Rep 2024;73:1–32. 10.15585/mmwr.rr7301a1 PMC10849099 38319847

[28] EI Richmond M Hoang W Shuel M. Laboratory evaluation of the Chembio DPP Syphilis Screen & Confirm point-of-care test on serum and simulated blood samples. J Assoc Med Microbiol Infect Dis Can 2024;9:82–94. 40641811 10.3138/jammi-2023-0035 PMC12169436

[29] Vrbová E Mikalová L Grillová L. A retrospective study on nested PCR detection of syphilis treponemes in clinical samples: PCR detection contributes to the diagnosis of syphilis in patients with seronegative and serodiscrepant results. PLoS One 2020;15:e0237949. 32817658 10.1371/journal.pone.0237949 PMC7446855

[30] Kamb ML Newman LM Riley PL. A road map for the global elimination of congenital syphilis. Obstet Gynecol Int 2010;2010:312798. 20706693 10.1155/2010/312798 PMC2913802

