# Supplementary material for: The Potential for Combined Treponemal/Nontreponemal Rapid Point-of-Care Test and Treponema pallidum Polymerase Chain Reaction in the Diagnosis of Gestational and Congenital Syphilis in a Low-Resource, High-Prevalence Setting: Pilot Data From Malawi
Source: Sex Transm Dis. 2026 May 15;53(8):510–7. doi: 10.1097/OLQ.0000000000002356 (PMC13326932; doi:10.1097/OLQ.0000000000002356)
Supplement: Supplementary file 1 [file std-53-510-s001.pdf]

Supplemental Digital Content 1

Supplementary methods: Maternal and Congenital Syphilis Case Definitions

**Maternal Syphilis staging**	
Early Syphilis	Negative treponemal test at ANC1 and positive treponemal test or RPR at delivery
Late or unknown stage syphilis	Positive treponemal test at ANC1 and positive treponemal test or RPR at delivery
**Infant case definitions**	
CDC Confirmed or probable congenital syphilis	a. An abnormal physical examination consistent with CS, in a syphilis exposed infant AND/OR<br>b. Syphilis PCR positive from infant secretions AND/OR<br>b. Infant RPR $\geq$ 4 fold maternal RPR
CDC Possible congenital syphilis *	a. Infant RPR <4 fold maternal RPR at delivery AND<br>b. Maternal early untreated syphilis as above without 1 dose of BPG > 30 days pre delivery OR<br>c. Maternal late untreated syphilis as above without 3 doses of BPG > 30 days pre delivery
CDC Congenital Syphilis less likely	a. Normal physical examination AND<br>b. Infant RPR <4 fold maternal RPR at delivery AND<br>c. *Maternal syphilis with treatment appropriate for stage, ie* 3 doses of BPG > 30 days pre delivery where stage is undetermined.
WHO CS requiring treatment	Incomplete maternal treatment history of three doses of IM Benzathine Penicillin in this pregnancy at least 30 days prior to delivery

Supplemental Digital Content 1: Criteria applied to (i) stage syphilis in pregnancy, and for (ii) infant risk assessment based on CDC and WHO guidance.
ANC1; First antenatal Care attendance, BPG; Benzathine Penicillin G, CDC; Centre for Disease Control, CS;Congenital Syphilis, RPR; Reactive Plasma Reagin, WHO; World Health Organisation. **Maternal syphilis without treatment appropriate for stage. Where stage is unknown, 3 doses of BPG (C) is deemed to be the only adequate treatment in pregnancy*
