# Supplementary material for: The Potential for Combined Treponemal/Nontreponemal Rapid Point-of-Care Test and Treponema pallidum Polymerase Chain Reaction in the Diagnosis of Gestational and Congenital Syphilis in a Low-Resource, High-Prevalence Setting: Pilot Data From Malawi
Source: Sex Transm Dis. 2026 May 15;53(8):510–7. doi: 10.1097/OLQ.0000000000002356 (PMC13326932; doi:10.1097/OLQ.0000000000002356)
Supplement: Supplementary file 2 [file std-53-510-s002.pdf]

Supplemental digital content 5

Summary of results for infants with confirmed CS by positive nasopharyngeal PCR.

Symptoms	CT value of positive NP PCR swab	Maternal RPR	Infant RPR	Fold change in paired RPR	Maternal Dual RDT	Infant Dual RDT
Congenital syphilis	29.923	1:4	1:8	2- fold rise	TT+/NTT+	TT+/NTT+
Sepsis	31.718	1:8	1:16	2-fold rise	TT+/NTT+	TT+/NTT+
HIE	28.608	1:8	1:4	2-fold fall	TT+/NTT+	TT+/NTT+
Premature	26.639	1:8	N/D	N/A	TT+/NTT-	N/D

Supplemental Digital Content 5 **-** Summary of results for infants who tested positive by *T. pallidum* PCR on nasopharyngeal swab, highlighting the potential importance of PCR testing in congenital syphilis. (CS; Congenital Syphilis, HIE; Hypoxic Ischaemic Encephalopathy, RPR Rapid Plasma Reagin, PCR; Polymerase Chain Reaction, NTT+; non-treponemal test band positive, NTT-; non-treponemal test band negative, TT+; treponemal test band positive, TT-; treponemal test band negative, T-RDT; Treponemal Rapid Diagnostic Test)
