# Supplementary material for: The Potential for Combined Treponemal/Nontreponemal Rapid Point-of-Care Test and Treponema pallidum Polymerase Chain Reaction in the Diagnosis of Gestational and Congenital Syphilis in a Low-Resource, High-Prevalence Setting: Pilot Data From Malawi
Source: Sex Transm Dis. 2026 May 15;53(8):510–7. doi: 10.1097/OLQ.0000000000002356 (PMC13326932; doi:10.1097/OLQ.0000000000002356)
Supplement: Supplementary file 3 [file std-53-510-s003.pdf]

Supplemental Digital Content 3

Dual RDT in the monitoring of maternal response to treatment

	Maternal DPP® Screen and Confirm at enrolment	Maternal RPR at enrolment	Maternal DPP® Screen and Confirm at follow up	Maternal RPR at follow-up	Maternal treatment post delivery
**Rise in RPR**	TT+ / NTT+	1:2	TT+ / NTT+	1:4	1 dose IM BPG
	TT+ / NTT+	1:2	TT+ / NTT -	1:8	1 dose IM BPG
	TT+ / NTT+	1:2	TT+ / NTT+	1:8	1 dose IM BPG
	TT+ / NTT+	1:2	TT+ / NTT+	1:4	1 dose IM BPG
	TT+ / NTT+	1:2	TT+ / NTT+	1:4	1 dose IM BPG
	TT+ / NTT+	1:2	TT+ / NTT+	1:4	1 dose IM BPG
**RPR unchanged**	TT+ / NTT+	1:8	TT+ / NTT+	1:8	1 dose IM BPG
	TT+ / NTT -	1:8	TT+ / NTT+	1:8	1 dose IM BPG
	TT+ / NTT+	1:2	TT+ / NTT -	1:2	1 dose IM BPG
	TT+ / NTT+	1:4	TT+ / NTT+	1:4	1 dose IM BPG
	TT+ / NTT+	1:8	TT+ / NTT+	1:8	1 dose IM BPG
	TT+ / NTT -	Negative	TT+ / NTT -	Negative	1 dose IM BPG
**Falling RPR**	TT- / NTT+	1:4	TT- / NTT -	Negative	1 dose IM BPG
	TT+ / NTT+	1:2	TT+ / NTT+	Negative	1 dose IM BPG
	TT- / NTT+	1:2	TT- / NTT-	Negative	1 dose IM BPG
	TT+ / NTT+	1:4	TT+ / NTT+	Negative	1 dose IM BPG
	TT+ / NTT+	1:2	TT+ / NTT+	Positive	1 dose IM BPG
	TT+ / NTT -	1:2	TT+ / NTT+	Negative	1 dose IM BPG

Supplemental Digital Content 3 – Table outlining paired maternal RPR and Dual RDT to monitor infant response to treatment. Post-natal treatment data, where known, is documented.
(BPG; benzathine penicillin, IM; intra-muscular, NTT+; non-treponemal test band positive, NTT-; non-treponemal test band negative, TT+; treponemal test band positive, TT-; treponemal test band negative)
