# Supplementary material for: The Potential for Combined Treponemal/Nontreponemal Rapid Point-of-Care Test and Treponema pallidum Polymerase Chain Reaction in the Diagnosis of Gestational and Congenital Syphilis in a Low-Resource, High-Prevalence Setting: Pilot Data From Malawi
Source: Sex Transm Dis. 2026 May 15;53(8):510–7. doi: 10.1097/OLQ.0000000000002356 (PMC13326932; doi:10.1097/OLQ.0000000000002356)
Supplement: Supplementary file 4 [file std-53-510-s004.pdf]

Supplemental Digital Content 4

Dual RDT to Monitor Infant Response to antenatal and post-natal treatment.

Infant category	Dual RDT at birth	Infant RPR at birth	Infant Treatment	Dual RDT at follow-up	Infant RPR at follow-up
**Syphilis exposed, inadequate antenatal treatment**	TT+ / NTT-	1:16	-	TT+ / NTT+	1:4
	TT+ / NTT+	1:4	IV benzyl-penicillin	TT+ / NTT+	1:2
	TT+ / NTT-	1:4	-	TT+ / NTT+	Negative
	TT+ / NTT-	1:4	-	TT+ / NTT+	1:2
	TT+ / NTT-	Negative	-	TT+ / NTT+	Negative
	TT- / NTT -	Negative	-	TT+ / NTT+	Negative
	TT- / NTT -	Negative	-	TT- / NTT -	Negative
	TT- / NTT -	Negative	-	TT- / NTT -	Negative
	TT- / NTT -	Negative	-	TT- / NTT -	Negative
	TT- / NTT -	Negative	-	TT- / NTT -	Negative
**Syphilis exposed, adequate antenatal treatment**	TT+ / NTT-	1:2	-	TT+ / NTT-	Negative
	TT+ / NTT-	Negative	-	TT+ / NTT-	Negative
	TT+ / NTT-	Negative	-	TT- / NTT+	1:2

Supplemental Digital Content 4 – Table outlining paired infant RPR and Dual RDT to monitor infant response to treatment. (IV; intravenous, NTT+; non-treponemal test band positive, NTT-; non-treponemal test band negative, TT+; treponemal test band positive, TT-; treponemal test band negative)
