# Supplementary material for: The Potential for Combined Treponemal/Nontreponemal Rapid Point-of-Care Test and Treponema pallidum Polymerase Chain Reaction in the Diagnosis of Gestational and Congenital Syphilis in a Low-Resource, High-Prevalence Setting: Pilot Data From Malawi
Source: Sex Transm Dis. 2026 May 15;53(8):510–7. doi: 10.1097/OLQ.0000000000002356 (PMC13326932; doi:10.1097/OLQ.0000000000002356)
Supplement: Supplementary file 5 [file std-53-510-s005.pdf]

Supplemental Digital Content 6

Near-patient diagnostics (Dual RDT) alone in infants for the risk stratification of CS.

**Infant Dual RDT result:**	**'Higher risk'**<br><br>**Syphilis exposed infants**<br><br>**RPR positive any titre**<br>**and/or**<br>**NP PCR positive**<br>**and/or**<br>**symptomatic CS**<br><br>*N=19 with paired bloods available*	**'Lower risk'**<br><br>**Syphilis exposed infants**<br><br>**RPR negative**<br>**and**<br>**NP PCR negative or not done**<br>**and**<br>**No symptoms of CS**<br><br>*N=17 with paired bloods available*
**Infant TT+/NTT+**	6	0
**Infant TT+/NTT-**	12	8
**Infant TT-/NTT+**	1	0
**Infant TT-/NTT-**	0	9

Supplemental Digital Content 6: Application of the Dual RDT alone in the risk assessment of infants compared to gold standard diagnostics (RPR, PCR) and clinical assessment. This analysis was performed independent of maternal treatment history, to syphilis exposed infants. Infant syphilis exposure was defined based on maternal (i) RPR positivity (ii) positive TT band on antenatal T-RDT or (iii) positive TT band on Dual RDT.
(PCR; polymerase chain reaction, RPR; Rapid Plasma Reagin, T-RDT; Treponemal Rapid Diagnostic Test, TT+; treponemal test band positive, NTT+ non-treponemal test band positive, TT-; treponemal test band negative, NTT- non-treponemal test band negative)
