# Supplementary material for: The Potential for Combined Treponemal/Nontreponemal Rapid Point-of-Care Test and Treponema pallidum Polymerase Chain Reaction in the Diagnosis of Gestational and Congenital Syphilis in a Low-Resource, High-Prevalence Setting: Pilot Data From Malawi
Source: Sex Transm Dis. 2026 May 15;53(8):510–7. doi: 10.1097/OLQ.0000000000002356 (PMC13326932; doi:10.1097/OLQ.0000000000002356)
Supplement: Supplementary file 6 [file std-53-510-s006.pdf]

Supplemental Digital Content 7

Near patient diagnostics (Dual RDT) on paired maternal and infant samples in the risk stratification of CS.

	**Total**	**CS diagnosis +ve confirmed/probable**	**CS diagnosis possible**	**CS diagnosis less -likely**
**All infants 'syphilis-exposed' with positive maternal RPR**	**Maternal RPR+<br>Infant RPR+<br>AND<br>Any maternal treatment**	**Infant PCR+<br>OR<br>Infant RPR at least four-fold or greater that of mother<br>OR<br>Symptoms of CS**	**Infant RPR ≤ four-fold that of mother<br>AND<br>PCR negative or not done<br>AND<br>No symptoms of CS<br>AND<br>Mother received 3 doses of IM BPG ≤30 days before delivery**	**Syphilis exposed infants born to mothers who received 3 doses of IM BPG ≥30 days before delivery<br>AND<br>infant RPR ≤ four-fold that of mother**
**Total**	**19**	**6**	**11**	**2**
**Dual RDT result**				
Maternal TT+/NTT-<br>Infant TT+/NTT+	**0**	0	0	0
Maternal TT+/NTT+<br>Infant TT+/NTT+	**6**	3	3	0
Maternal TT+/NTT+<br>Infant TT+/NTT-	**8**	2	5	1
Maternal TT+/NTT-<br>Infant TT+/NTT-	**4**	0	3	1
Maternal TT-/NTT+<br>Infant TT-/NTT+	**1**	1	0	0

Supplemental Digital Content 7- Application of paired maternal and infant DPP® Syphilis Screen & Confirm in the risk stratification of infant CS. (PCR; polymerase chain reaction, RPR; Rapid Plasma Reagin, TT+ve; treponemal test band positive, NTT+ve non-treponemal test band positive, TT-ve; treponemal test band negative, NTT-ve non-treponemal test band negative)
