# Supplementary material for: The Potential for Combined Treponemal/Nontreponemal Rapid Point-of-Care Test and Treponema pallidum Polymerase Chain Reaction in the Diagnosis of Gestational and Congenital Syphilis in a Low-Resource, High-Prevalence Setting: Pilot Data From Malawi
Source: Sex Transm Dis. 2026 May 15;53(8):510–7. doi: 10.1097/OLQ.0000000000002356 (PMC13326932; doi:10.1097/OLQ.0000000000002356)
Supplement: Supplementary file 7 [file std-53-510-s007.pdf]

Supplemental Digital Content 8

Venn diagrams presenting the results of (i) treponemal band (ii) non-treponemal bands on Dual RDT and (iii) nasopharyngeal PCR in 'higher risk' and 'lower risk' infants.

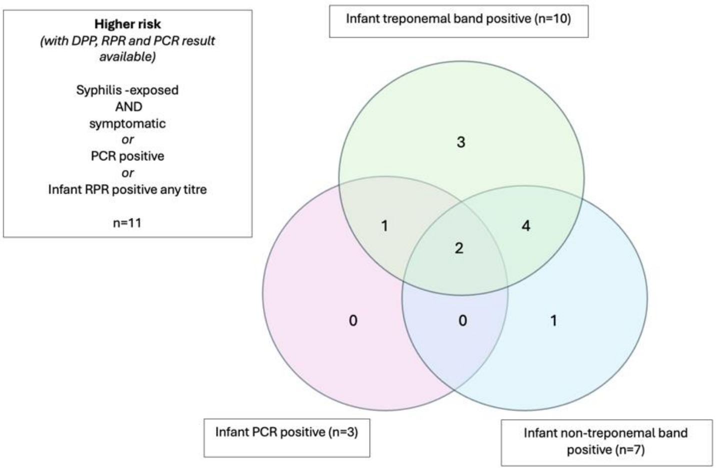

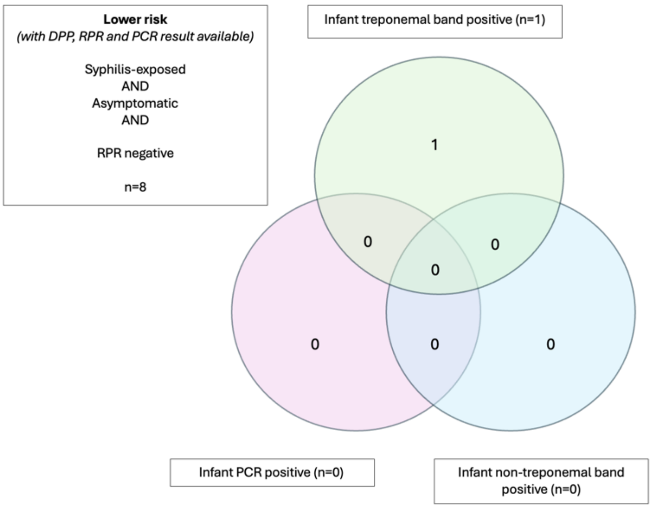

Supplemental Digital Content 8 - Syphilis exposed infants with available Dual RDT result an PCR were stratified into higher-risk (11/23; infants born to inadequately treated mothers and infant RPR positive +/- PCR positive) and lower-risk groups (infants born to adequately treated mothers with infant RPR and PCR negative). The definitions of risk below are roughly equal to CDC confirmed, probable and possible ('higher risk') and CDC less likely ('lower risk'). No 'CDC unlikely' infants were included as this cohort did not have access to regular RPR monitoring during pregnancy. Four non syphilis-exposed infants were removed from this sub-analysis.

CDC; Centre for Disease Control, PCR; polymerase chain reaction, RPR; Rapid Plasma Reagin, T-RDT; Treponemal Rapid Diagnostic Test.
